# Web alert: Microbes and plant nutrients: An annotated selection of World Wide Web sites relevant to the topics in *microbial biotechnology*

**DOI:** 10.1111/1751-7915.14275

**Published:** 2023-05-27

**Authors:** Lawrence P. Wackett, McKnight

**Affiliations:** ^1^ Department of Biochemistry, Molecular Biology & Biophysics BioTechnology Institute, University of Minnesota St. Paul Minnesota USA

Plant microbiomes

https://allianceforscience.org/blog/2022/04/plant‐microbiome‐key‐to‐weaning‐agriculture‐off‐chemical‐inputs/

Just as research on the human gut microbiome shows linkages to health of people, studies of plant microbiomes are beginning to provide insights into engineering healthy microbiomes for plants.

NextGen fertilizer challenge

https://www.epa.gov/innovation/next‐gen‐fertilizer‐challenges

While not specifically on microbes, this workshop involved many stakeholders in seeking better ways to provide nutrients to plants, including microbial‐based formulations.

Meta‐analysis of biostimulants

https://www.frontiersin.org/articles/10.3389/fpls.2022.836702/full

This study comprehensively examined the efficacy of biostimulants across crop plants, conditions of use, environment, and economic value.

Plant soil nutrient uptake

https://www.nature.com/scitable/knowledge/library/plant‐soil‐interactions‐nutrient‐uptake‐105289112/

This *Nature* knowledge web page focused on plant uptake of nutrients, but also has a section on microbial association with roots, particularly mycorrhizal fungi.

Rhizosphere roots and microbes

https://www.nature.com/scitable/knowledge/library/the‐rhizosphere‐roots‐soil‐and‐67500617/

This *Nature* knowledge web page focused on the rhizosphere, or the soil root zone, particularly regarding the type of microbes often active in that zone.

Rhizosphere bacteria compendium

https://www.sciencedirect.com/topics/agricultural‐and‐biological‐sciences/rhizosphere‐bacteria

This compendium of resources on rhizosphere bacteria includes books, book chapters and journal articles.

Microbial enhancement of nutrients

https://link.springer.com/article/10.1007/s44154‐021‐00027‐w

This review provides a good overview of how bacteria and fungi aid in the mobilization of many nutrients for plant root acquisition.

Plants recruiting microbes

https://www.rutgers.edu/news/how‐plants‐harness‐microbes‐get‐nutrients

This article describes a paper purporting that plants may attract microbes, where they enter roots and are lysed to release needed nutrients for the plant.

Phosphate mobilization

https://journals.asm.org/doi/10.1128/spectrum.00290‐22

Phosphate is abundant but often bound in mineral and organic forms that plants cannot access. This study examined the types of bacteria that can liberate the nutrient from mineral phosphate and phytic acid.

Sulfur material cycling

https://pubs.usgs.gov/of/2002/of02‐298/of02‐298.pdf

Sulfur is an important nutrient for all living things. This report from the U.S. Geological Survey describes the flow of sulfur via natural and anthropogenic processes.

Micronutrients

https://www.nature.com/articles/s41522‐022‐00363‐3

This study used data from hundreds of agricultural fields to better understand the importance of micronutrients in shaping soil microbial communities.

